# FROM THE NATHAN SHOCK CENTERS COORDINATING CENTER: BIOLOGY OF AGING FOR NONBIOLOGISTS

**DOI:** 10.1093/geroni/igae098.1906

**Published:** 2024-12-31

**Authors:** Odette van der Willik, Steven Austad

**Affiliations:** Chair; Co-Chair

## Abstract

Advances in the biology of aging that promise to dramatically extend the healthy length of human life are now appearing at a breathtaking rate. At the same time, baseless claims of research breakthroughs in maintaining youth are everywhere in the news. It is often difficult for people outside basic aging research to keep track of what is real and what is not. This symposium brings together scientists working in some of the most exciting research areas to update attendees in terms that will be understandable to anyone. Our speakers were selected both for their scientific achievements and for their outstanding skill as science communicators. Dr. Passos will discuss the rapidly expanding field of senotherapeutics to identify drugs that can eliminate senescent cells (senolytics) and what is needed in the development of therapies that target cellular aging. Dr. Molina will review the development of promising biomarkers of human aging and will provide examples of how markers are being tested and applied in a range of clinical studies. Dr. Panda will provide insights from circadian rhythm science and how new lifestyle choices have the potential to be implemented on a population scale. And Dr. Espinoza will discuss the basic principles of geroscience and the use of such therapies toward the goal of improving healthspan (as opposed to the traditional single disease approach). Dr. Valenzano will update us on how important the microbes that live in and on us are turning out to be to our later life health.

